# Magnetic resonance imaging of temporomandibular joint with anterior disk dislocation without reposition - long-term results

**DOI:** 10.1007/s00784-016-1800-9

**Published:** 2016-04-16

**Authors:** M. Bristela, M. Schmid-Schwap, J. Eder, G. Reichenberg, M. Kundi, E. Piehslinger, S. Robinson

**Affiliations:** 1 Department of Fixed and Removable Prosthodontics, University Clinic of Dentistry, Sensengasse 2a, 1090 Vienna, Austria; 2 Dental Office, Vienna, Austria; 3 Institute of Environmental Health, Center for Public Health, Medical University of Vienna, Kinderspitalgasse 15, 1090 Wien, Austria; 4 Diagnostic Center Urania, Laurenzerberg 2, 1010 Vienna, Austria

**Keywords:** MRI, TMD, Pseudo-disk, Disk position, Disk displacement without reposition

## Abstract

**Objectives:**

Anterior disk dislocation (ADD) without reposition in the temporomandibular joint (TMJ) may be associated with morphological changes in the retrodiscal region of the bilaminar zone presenting as pseudo-disk (PD). The present study was initiated to investigate the development of retrodiscal fibrosis in a period of 4–8 years and to assess if patients with a PD show differences in the clinical and radiologic findings versus patients without a PD.

**Materials and Methods:**

In a retrospective follow-up study of 33 consecutive patients with ADD without reposition in one or both TMJs, a clinical and MRI-supported evaluation was conducted 4 to 8 years after baseline diagnosis.

**Results:**

In 45 % of the TMJs with ADD without reposition, a PD could be identified. Twenty-one of 31 patients who showed pain at the baseline examination (VAS mean 56 ± 38) were pain free. The mouth opening capacity (MO) of the mandible could be increased in 80 %. There were no statistical significant differences between patients with or without PD in these clinical features. The MRI parameters effusion and translation showed a statistical tendency for more improvement in the group with PD (*p* = 0.061, 0.064).

**Conclusion:**

In about half of the patients, a structure corresponding to a pseudo-disk developed during follow-up. Pain and the mouth opening capacity improved in all patients independent of the development of a PD.

**Clinical Relevance:**

Detection of a PD during follow-up of patients with ADD without spontaneous reposition does neither predict favorable nor worse therapy response and clinical course.

## Introduction

“Temporomandibular disorder” (TMD) stands for a wide range of clinical articular and muscular problems in the orofacial region. The clinical complex of symptoms comprising articular noises, ear pain, and/or headache, frequently associated with tinnitus, dizziness, and burning tongue, was initially described by Costen and termed Costen’s syndrome (Costen et al. 1934).

Clinical symptomatology can be characterized by pain, tenderness on palpation of the temporomandibular joints (TMJ) and masticatory muscles, articular noises, and irregular or restricted jaw function [1, 2]. Approximately one third of the adult population shows at least one symptom of TMD [3].

TMD can be subdivided into arthrogenic and myogenic disorders [4]. Disk dislocation of the TMJ is classified as an arthrogenic disorder and categorized according to the displacement plane. Displacement of the disk in the sagittal plane is termed anterior or posterior displacement, while displacement in the transversal plane is termed medial or lateral.

Magnetic resonance imaging (MRI) of the anatomical structures has been established as internationally recognized standard for the assessment of position, morphology, and mobility of disk and condyle and evaluation of adjacent structures [3]. In this respect, three Tesla units appear superior to those with a lower field strength using the same examination time [5, 6].

By definition, anterior disk dislocation (ADD) is present, when the posterior border of the disk is visualized in MRI anterior to the 12 o’clock position of the condyle [7–9]. Moreover, disk position will depend on the inclination of the eminence and ventral positioning of the disk will increase with the inclination of the eminence [10]. For therapeutic management, it is relevant to assess disk mobility during mouth opening (MO). If the disk resumes its physiological position on the condyle during the jaw opening movement, this is termed ADD with reposition. However, if the articular disk remains dislocated in front of the condyle during the complete opening movement, this is termed as ADD without reposition [11]. This may result in a restriction of movement (locked joint). In 9 % of temporomandibular joints with ADD with reposition, this condition will develop to ADD without reposition within a period of 3 years [12].

In MRI, the chronically displaced articular disk mostly shows a very low signal intensity and frequently a flat shape. Its posterior portion and the bilaminar zone render a lower signal than in the healthy TMJ [13]. Histologically, increased fibroblast activity, neogenesis of collagen fiber bundles, and hyalinization can be demonstrated in the TMJ with ADD without reposition [14–16]. Some authors postulate that chronic inappropriate dorsal or dorsocranial loading of the bilaminar zone can result in the formation of a pseudo-disk [3, 13, 16, 17]. The histological studies of Scapino and the MRI evaluations of Katzberg show that fibrous cords from the bilaminar zone may occasionally form a “pseudo-disk”.

As yet, no precise or specific data have been reported regarding the time course required for this remodeling. However, the time passed since the disk displacement may also play a major role in explaining the variability of morphologic changes.

The present study was initiated to investigate the development of retrodiscal fibrosis, a so-called pseudo-disk, in a period of 4–8 years and to assess if patients with a pseudo-disk show differences in the clinical and radiologic findings versus patients without a pseudo-disk.

## Material and methods

The present study was approved by the Ethics Committee of the Medical University Vienna (EK 1742/2012). The investigations and procedures were performed after having obtained written informed consent of each participating patient.

Over the period from 2004 to 2008, more than 2300 patients were evaluated at the outpatient unit for temporomandibular disorders of the prosthetic department of the Dental University Clinic of Vienna with part of these patients undergoing MRI with three Tesla.

Fifty-one patients meeting the inclusion criteria (ADD without reposition, having had their baseline diagnostic evaluation at least 4 years before and MRI with three Tesla at the Diagnostic Center Urania) were invited for a follow-up examination. Exclusion criteria were patient’s age below 18 years, orthodontic treatment, tremor, presence of ferromagnetic objects or biostimulators, and pregnancy.

Out of the 51 patients included, 15 patients did not respond to contact by postal mail or phone and two patients refused any additional MRI. Thirty-four patients consented to participate and underwent the follow-up clinical evaluation. One of these patients did not present for the MRI.

Thus, 33 persons (66 joints) were enrolled in the study. Twenty-four joints showed no ADD without reposition, therefore, 42 joints were investigated. Twenty-eight (85 %) of the patients were female and five patients (15 %) were male. The patients had an average age of 53 ± 18 years (range 23–77).

At the baseline examination, collection of the patient’s detailed history and evaluation of pain sensation were followed by a standardized clinical evaluation [8]. This evaluation comprises facial neurological assessment, evaluation of jaw movements, and palpation of muscles and temporomandibular joints as well as assessment of occlusion and articulation.

Pain sensation was evaluated using the Visual Analog Scale (VAS). The range between no pain and maximum pain is displayed by a color line with increasing color intensity. Using a movable bar, the patient is asked to indicate his/her pain experienced at rest, under function (e.g., chewing and mouth opening) and maximum pain experienced. On the backside of the scale, not visible for the patient, the respective numerical value (0–100) is displayed.

At the baseline visit, all patients with TMJ complaints received detailed information on the function and dysfunction of the TMJ as well as about pathogenic factors and got behavioral guidelines and information about the impact of habits (e.g., nail biting, gum chewing, and clenching during the day).

Two of the patients had received no therapy at all, two patients only splint therapy, four patients physiotherapy, and 25 patients a combination of both. Splints were fabricated following the concept of frontal and canine guidance and were adapted regularly over a period of 3 months. Physiotherapy was conducted by experienced therapists. A therapy-package included 10 units, with session lasting 45 min.

Prior to performance of follow-up MRI, the same clinical parameters as for the baseline examination were evaluated again.

### MRI scan

Baseline and follow-up MRI were performed at the same institute to ensure equal examination conditions. Patients were positioned supine, a Sense Flex-M coil was placed exactly over the temporomandibular joint. All MRI scans were done with a three Tesla unit of Philips Intera 3.0 (Philips Medical Systems, Netherlands) with closed mouth in parasagittal and paracoronal plane and with open mouth (defined jaw opening of 30 mm by means of bite jig) in parasagittal plane. The slice thickness of the images was 2 mm (Table 1). All images were evaluated by a radiologist specialized in head and neck MRI with 27 years of professional experience.

**Table 1:** Magnetic resonance imaging sequences

Magnetic resonance parameters of the sequences at 3.0 Tesla						
	T2w-TSE sequence		T1w-TSE sequence		PDw-TSE sequence	
	Parasagittal closed mouth		Paracoronal closed mouth		Parasagittal opened mouth	
	Baseline MRI	Follow-up MRI	Baseline MRI	Follow-up MRI	Baseline MRI	Follow-up MRI
	3.0	3.0	3.0	3.0	3.0	3.0
FOV	150	150	130	130	150	150
Slices	15	15	8	8	15	15
Slices thickness (mm)	2	2	2	2	2	2
TSE factor	14	14	4	6	6	6
TR (ms)	4823	4147	550	551	2,451	2.375
TE (ms)	80	80	13,9	8,4	20	20
Flip angle	90	90	90	90	90	90
NSA	4	2	3	4	4	2
Scan duration (min)	03:56	03:27	03:39	04:06	04:10	04:35
Measured voxel size (mm)	0.55/0.71/2.00	0.55/0.71/2.00	0.54/0.68/2.00	0.54/0.68/2.00	0.55/0.79/2.00	0.55/0.7/2.00
Reconstructed voxel size (mm)	0.29/0.29/2.00	0.29/0.29/2.00	0.51/0.5/2.00	0.51/0.50/2.00	0.29/0.29/2.00	0.29/0.29/2.00

*FOV* field of view, *TSE* turbo spin echo, *TR* time of repetition, *TE* echo time, *NSA* number of signals averaged,

For the assessment of morphological changes in the parasagittal plane of the joint, the central images were selected, in which the structures were best visible. The ancillary target criteria of radiologic evaluation are shown in Table 2.

**Table 2:** Ancillary target criteria of radiologic evaluation

Disk position	Anterior	Anterior-medial	Anterior-lateral		
Disk shape	Biconcave	Flat	Biconvex	Deformed	Disk residues
Signal intensity	Homogenous	Focal hyperintensities			
Inclination of eminence	Normal	Steep	Flat		
Translation	Normal	Restricted	None		
Effusion	Yes	No			
Condyle degeneration	Flattened	Cuspidated	Sclerotic	Disk remnants	
Condyle position	Distraction	Compression	Anterior	Lateral	Retral

### Statistical analysis

In order to ensure that the prerequisite for the independence of the observations is not violated, only one joint per patient was included in the evaluation. Thus, the number of temporomandibular joints evaluated was 33. Only the joint showing no pseudo-disk in the baseline evaluation was included in the analysis for those patients with bilateral joint involvement. If no pseudo-disk was seen in either the baseline or the follow-up evaluation, one of the two joints was randomly selected.

Statistical evaluation involved the assessment to which extent clinical and radiologic parameters in the baseline evaluation allowed for a prognosis or prediction of the development of a pseudo-disk as well as the question whether patients developing a pseudo-disk during the follow-up period could be differentiated with regard to the development of clinical and radiologic findings. The first question was assessed using logistic regression. The second question was tested using a generalized linear model with repeated measurements assuming a binomial, multinomial, or normal distribution depending on variable type. Development of a pseudo-disk in clinical course was included as group factor. For all analyses, which were performed using SPSS 22.0 (IBM, USA), a *p* value below 0.05 was taken as significance level and a *p* value below 0.1 was considered a statistical tendency.

## Results

**Fig. 1:**
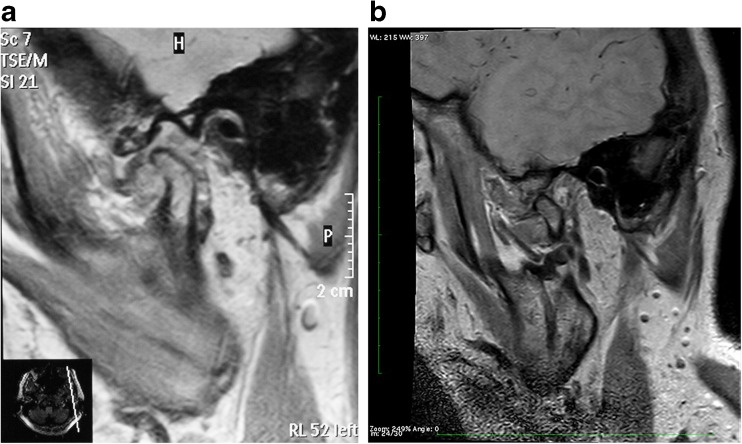
**a** 28.12.2006: Parasagittal PD-sequence with open mouth in a patient with anterior disk dislocation without reposition and more severely impaired translation (condyle remains posterior to the articular eminence). **b** 14.10.2013: Parasagittal PD-sequence with open mouth 7 years later shows a faint pseudo-disk and range of mouth opening has slightly improved

**Fig. 2:**
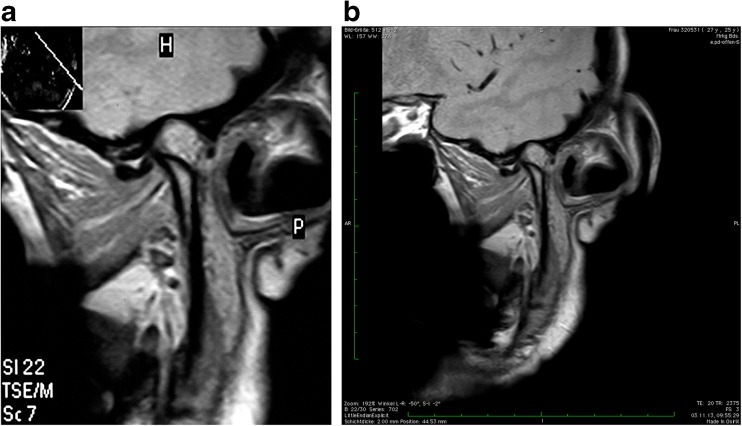
**a** 24.7.2008: Parasagittal PD-sequence with open mouth in a patient with anterior disk dislocation without reposition **b** 3.11.2013: Parasagittal PD-sequence with open mouth five years later without pseudodisk

In nine of the 33 patients included, a disk displacement without reposition could be noted in both temporomandibular joints. In 24 patients, an ADD without reposition could only be shown in one joint in the MRI. In the baseline MRI scan, two patients already showed morphological changes in the form of a pseudo-disk in one joint. In the follow-up scan after 4–8 years, a pseudo-disk was found in 19 of the 42 temporomandibular joints (Fig. 1a,b), while 23 joints did not show any pseudo-disk (Fig. 2a,b). The average age of the group without PD was 50 ± 17 years that of the group with PD 56 ± 17 years (*p* = 0.370).

At the time of the baseline examination, two patients were pain free (one patient in each of the groups with and without pseudo-disk). At the time of the follow-up evaluation, 23 patients were without pain, while 10 patients described pain. In the group without pseudo-disk, 12 patients (71 %) were pain free, and in the group with pseudo-disk, there were 11 (69 %).

**Fig. 3:**
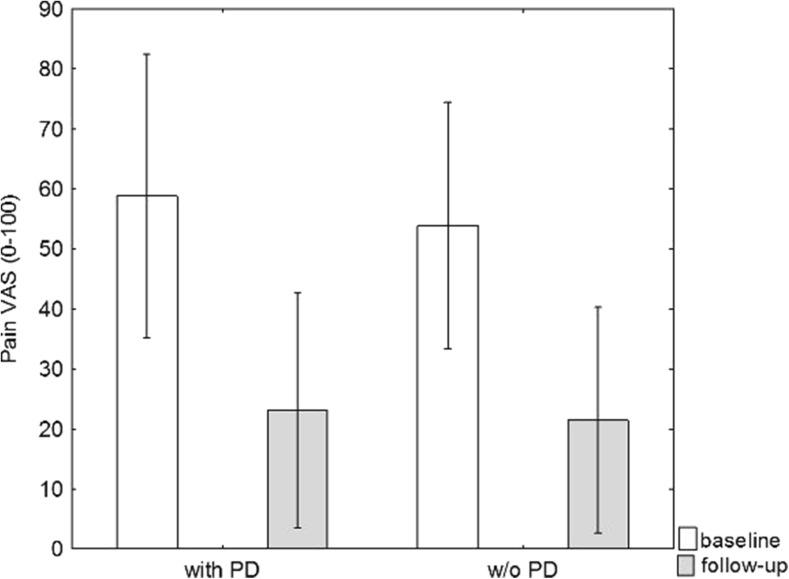
Pain visual analog scale (VAS) at baseline and follow-up. Mean and 95 % confidence intervals

Pain sensation on the VAS at the baseline evaluation was on average 56 ± 38 mm (range 0–100); 54 ± 39 mm in the group without PD; and 59 ± 39 mm in the group with PD. At the follow-up, a continued decrease could be seen: 22 ± 36 mm (range 0–94); 21 ± 37 mm in the group without PD; and 23 ± 37 mm in that with PD. These improvements were statistically significant regardless of the presence of PD (*p* < 0.001) (Fig. 3).

In the baseline evaluation, 20 of the 33 TMJs showed a purely anterior displacement of the articular disk, one patient presented with an anterior-medial displacement and in 12 patients an anterior-lateral displacement was seen. At the follow-up evaluation, 16 of the 33 TMJs showed a purely anterior displacement of the articular disk, 5 TMJs an anterior-medial displacement, and 12 an anterior-lateral displacement. No significant difference in the distribution of disk position between patients with and without PD was seen in the baseline and the follow-up. Position and morphology of the condyle showed no statistical significant differences between the two groups. Disk shape at baseline evaluation was biconcave in 16 of the 33 TMJs evaluated, flat in 17 TMJs; no disk deformation or disk residues could be seen in any of the TMJs evaluated. Regarding disk shape at the time of the follow-up evaluation, it was biconcave in 15 of the 33 TMJs, flat in 15 and showed deformation in one TMJ of the group without PD; in two TMJs only disk residues could be seen. Neither the patients without PD nor those with PD showed any differences between baseline and follow-up evaluation. Twenty-five of the 33 TMJs showed homogenous disk signal intensity at the time of the baseline evaluation, while eight disks showed focal hyperintensities. At the time of the follow-up evaluation, 23 of the 33 TMJs had homogenous disk signal intensity, while focal hyperintensities were seen in 10 disks. At the time of the baseline evaluation, 12 of the 33 TMJs showed a normal eminence, while 16 TMJs had a steep and five had a flat eminence. At the follow-up evaluation, 10 TMJs had a normal eminence, 13 had a steep and 10 had a flat eminence. No significant correlation was seen between the inclination of the eminence and the occurrence of a pseudo-disk. Of the 33 TMJs, four showed normal translational movement at the time of the baseline evaluation, while 28 TMJs showed restricted translational movement and one TMJ no translational movement at all. At the time of the follow-up evaluation, eight TMJs showed normal translational movement, 23 TMJs restricted translational movement and two no translational movement at all. The improvement of translation showed a statistical tendency in the group with PD (*p* = 0.064). There was no significant difference in translation between the two groups. At the time of the baseline evaluation, 17 TMJs had effusion, while no effusion was seen in 16 TMJs. At the follow-up evaluation, effusion was seen in 10 TMJs and no effusion was present in 23. A statistical tendency for a decrease of effusion was found in both groups (*p* = 0.088 group without PD, *p* = 0.061 group with PD), with no statistical significant difference between the two groups (Table 3).

**Table 3:** MRI parameters in joints with and without pseudo-disk (PD) in baseline and follow-up examinations

		W/o pseudo-disk			With pseudo-disk			
		Baseline	Follow-up	Comparison baseline vs. follow-up	Baseline	Follow-up	Comparison baseline vs. follow-up	Comparison of change (with vs. w/o PD)
Parameter	Category	*N* (%)	*N* (%)	*p* value	*N* (%)	*N* (%)	*p* value	*p* value
Disk position	Anterior	12 (71)	11 (65)	0.594	8 (50)	5 (31)	0.245	0.248
	Anterior/lateral	4 (24)	4 (24)		8 (50)	8 (50)		
	Anterior/medial	1 (6)	2 (12)		0 (0)	3 (19)		
Disk shape	Biconcave	8 (47)	6 (35)	0.365	8 (50)	9 (56)	0.337	0.383
	Flat	9 (53)	9 (53)		8 (50)	6 (38)		
	Deformation	0 (0)	1 (6)		0 (0)	0 (0)		
	Residuals	0 (0)	1 (6)		0 (0)	1 (6)		
Signal intensity	Focal	3 (18)	5 (29)	0.139	5 (31)	5 (31)	0.144	0.139
	Homogenous	14 (82)	12 (71)		11 (69)	11 (69)		
Slope eminence	Normal	7 (41)	6 (35)	0.971	5 (31)	4 (25)	0.834	0.671
	Steep	7 (41)	6 (35)		9 (56)	7 (44)		
	Flat	3 (18) 5	5 (29)		2 (13)	5 (31)		
Translation	Normal	1 (6)	2 (12)	0.443	3 (19)	6 (38)	0.064	0.195
	Normal	15 (88)	13 (76)		13 (81)	10 (63)		
	Normal	1 (6)	2 (12)		0 (0)	0 (0)		
Effusion		9 (53)	5 (29)	0.088	8 (50)	5 (31)	0.061	0.776
Condyle degeneration	Flattened	11 (65)	11 (65)	1.000	9 (56)	12 (75)	0.062	0.420
	Cuspidated	10 (59)	14 (82)	0.031	9 (56)	12 (75)	0.062	0.762
	Sclerotic	10 (59)	13 (76)	0.063	8 (50)	10 (63)	0.135	0.765
	Broken	0 (0)	1 (6)	0.995	1 (6)	2 (13)	0.569	1.000
Condyle position	Distraction	3 (18)	3 (18)	1.000	5 (31)	3 (19)	0.138	0.581
	Compression	1 (6)	1 (6)	1.000	0 (0)	0 (0)	1.000	1.000
	Anterior	0 (0)	0 (0)	1.000	0 (0)	2 (13)	0.786	1.000
	Lateral	1 (6)	2 (12)	0.314	2 (13)	4 (25)	0.318	0.955
	Retral	3 (18)	2 (12)	0.397	1 (6)	0 (0)	0.995	1.000

*P* values for comparison between baseline and follow-up in joints with and without PD and for comparison of change from baseline to follow-up between groups

Twenty-five patients (76 %) were treated with a combination of splint and physiotherapy, four patients (12 %) only received physiotherapy, two patients (6 %) only splint therapy, and two (6 %) no therapy at all.

The mean incisal edge distance before therapy was 41 ± 8 mm (range 27–61 mm); 41 ± 9 mm in the group without PD and 41 ± 6 mm in the group with PD. A significant increase of incisal edge distance was seen in the majority of participants in the follow-up evaluation. The mean distance was 46 ± 6 mm (range 33–60 mm) with 45 ± 7 mm in the group without and 46 ± 5 mm in the group without PD.

**Fig. 4:**
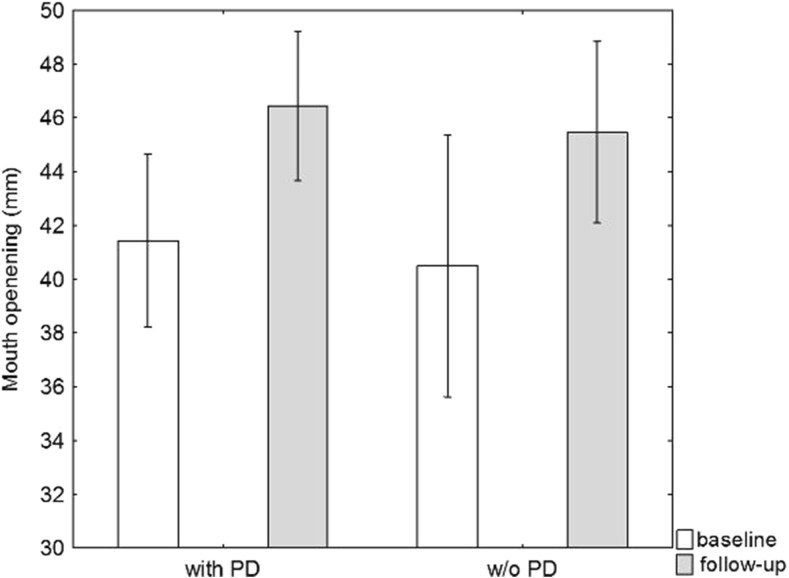
Mouth opening (mm) at baseline and follow-up. Mean and 95 % confidence intervals

The increase in mouth opening was statistically significant regardless of the presence of a PD (*p* < 0.001) (Fig. 4).

## Discussion

This historical prospective investigation of morphological changes in the temporomandibular joint is a clinical and MRI study of patients with anterior articular disk displacement without reposition. Most of the patients were treated with physiotherapy and/or splint therapy. The group without therapy in the present study was too small for demonstrating statistically relevant results. The follow-up evaluation covered a time range of 4–8 years. At the end of this period, the patients underwent a second clinical functional analysis and MRI scans. In 42 TMJs, 19 pseudo-disks were evaluated corresponding to a rate of almost 50 % with 17 of these pseudo-disks not seen in the baseline evaluation.

The use of higher field strengths in MRI and a dedicated coil has, compared to units with a field strength of 1.5 Tesla, allowed for improved imaging, especially for smaller joint structures [5, 6]. The present study protocol optimizes delineation of condyle, disk, bilaminar zone, and disco temporal and disco condylar joint compartments and the surrounding tissue [9].

According to Bumann, progressive adaptation of the bilaminar zone can be demonstrated in 70–90 % of all TMJs with a varying degree of disk displacement. He notes that this can only be determined with certainty in the MRI by simultaneous demonstration of low-signal structures behind the disk in T1- and T2-weighted images [18].

A marked inclination of the articular eminence has been indicated as etiological factor favoring temporomandibular dysfunction [19, 20]. As a result of the varying shape and inclination of the articular eminence, biomechanical components of the temporomandibular joint may change and thus play a key role in the development of an ADD [21]. The present study evaluated the inclination of the articular eminence, but no statistical significant correlation with pseudo-disk formation or pain could be found.

Previous reports have compared the symptoms of treated patients with ADD without reposition versus untreated patients showing no significant differences [12, 13]. In the present study, 25 patients (76 %) were treated with splint- and physiotherapy, four patients (12 %) received only physiotherapy, two patients (6 %) were treated with splint therapy only, and two patients (6 %) were given no therapy at all. The fact that some patients do not require any additional therapy following detailed information is consistent with the results of Orthlieb and McNeill who postulated that rapid pain relief using medication, appropriate information on behavioral measures may be sufficient in some patients to achieve appropriate pain elimination and, thus, adequate treatment success [22, 23]. Our patients were also adequately instructed during the baseline evaluation to employ appropriate measures in the case of obvious parafunctional activity and to avoid any hard or chewy food until appropriate improvement of symptoms. This is in line with the findings of Sato who described marked improvement of symptoms or complete absence of pain in 21 of the pain patients in a “follow-up” study after 27.1 months, although they did not undergo any treatment. However, the anterior displacement of the disk persisted [24, 25]. Thus, mobility of the disk appears to be critical for successful pain therapy [26, 27]. In the present study in the group with pseudo-disk, more joints showed normal translation than in the baseline evaluation. Also in the group without pseudo-disk, the number of joints with no translation as well as with normal translation increased.

With a total number of 33 joints studied and with most patients receiving the combination therapy recommended in literature [28–30], no statistically significant correlation between formation of a pseudo-disk and pain relief could be demonstrated. At the follow-up evaluation, 21 patients showed an improvement of pain symptoms and 25 patients of mouth opening and even the two patients without therapy were free of symptoms after a period of about 12 months. These results were consistent with those reported by other authors [23, 31].

McNeill described adaptation of the splint after approximately 6 weeks during the initial phase as unavoidable in order to account for the ongoing neuromuscular changes of the maxillomandibular relationship and to improve temporomandibular disorders. This will be done until a stable, reproducible intercuspation position and adequate pain relief has been achieved [23]. In the present study, the splints were also adapted at regular intervals over a period of several weeks.

Ohnuki compared the effects of different therapeutic approaches (manual manipulation, arthrocentesis, and arthroscopic surgery) in patients with ADD without reposition and showed that—regardless of the type of treatment used—ADD without reposition persisted in 90 % of the cases [26]. Similar results were reported after performing lavages in the TMJ, which were able to improve clinical symptoms, but could not correct the anterior disk displacement in most of the patients [24, 25, 32]. Even following arthroscopic surgery, disk position may frequently not be improved permanently [26, 27, 33]. A possible explanation for the general improvement of clinical symptoms may be the increased mobility of the articular disk following appropriate treatment [26]. Disk deformation in the TMJs treated with arthrocentesis and arthroscopic surgery was significantly higher than in patients treated with manual therapy or splint [26].

The particular strength of the present study includes the long time period of 4–8 years between the baseline and the follow-up evaluation of the patients as well as the exclusive use of the same dental and radiological center for all evaluations of the patients.

The number of study subjects must be considered as a shortcoming of the study, although for primary outcome variables of interest, pain and mouth opening, the study was sufficiently powered. As a result of the long time interval between the two evaluations, many patients eligible for inclusion could either not be contacted; on the other hand, only patients having undergone MRI scans with 3 Tesla at the baseline evaluation were enrolled in order to ensure comparability of the MRI images.

In summary, the present study could demonstrate a pseudo-disk in almost half of the temporomandibular joints with an ADD without reposition. This is consistent with the results of previous studies describing morphological changes manifesting as fibrosis and neovascularization in the presence of chronic ADD without reposition in the retrodiscal region of the bilaminar zone.

Of the 31 patients describing pain on palpation in the temporomandibular joint and in the preauricular region upon mouth opening and at rest, 21 patients were pain free at the follow-up evaluation and in 80 % of the patients significant improvement of mouth opening capacity could be achieved. Overall, the group with pseudo-disk showed similar results as the group without pseudo-disk. But there is some evidence that the improvement of the clinical and radiological parameters is more pronounced in the group with PD. Further prospective long-term studies should be performed to get more information about reparative processes of the temporomandibular joint with disk displacement without reposition.
